# Metastasis of advanced gastric cancer to the extraocular muscle: a case report

**DOI:** 10.1186/s13256-019-2031-x

**Published:** 2019-04-26

**Authors:** Sakiko Goto, Hiroaki Takeda, Yuriko Sasahara, Imi Takanashi, Hidetoshi Yamashita

**Affiliations:** 1 Department of Ophthalmology, Yamagata Prefectural Central Hospital, Yamagata, Japan; 2 Department of Ophthalmology, Yamagata University Faculty of Medicine, 2-2-2 Iida-nishi, Yamagata city, Yamagata 9909585 Japan; 3 Department of Internal Medicine, Yamagata Prefectural Central Hospital, Yamagata, Japan; 4 Department of Clinical Oncology, Yamagata Prefectural Central Hospital, Yamagata, Japan; 5 Department of Radiology, Yamagata Prefectural Central Hospital, Yamagata, Japan

**Keywords:** Extraocular muscle metastasis, Gastric cancer, Proptosis, Radiation therapy, Case report

## Abstract

**Background:**

Metastatic tumors in the orbit, especially from gastric cancer, are rare. We present a rare case of extraocular muscle metastasis from gastric cancer and raise consideration of metastasis to extraocular muscle as a differential diagnosis of proptosis/lid swelling in a patient with history of malignancy.

**Case presentation:**

A 54-year-old Japanese woman presented with proptosis, lid swelling, diplopia, and retro-orbital pain in her left eye, which she had been experiencing for 1 day. She had a medical history of poorly differentiated adenocarcinoma of the stomach, which had metastasized to several organs. A computed tomography scan showed enlargement of the medial rectus muscle in her left eye. She was diagnosed as having gastric cancer metastasis to the medial rectus muscle of her left eye, and received a total of 20 Gy radiation therapy to the orbit, which resulted in resolution of her ocular symptoms. She died 3 months after her initial visit to our ophthalmic department.

**Conclusions:**

Metastasis from malignancy should be considered in the differential diagnosis of a patient presenting with proptosis or lid swelling who has a history of gastric cancer. Radiation therapy of metastases in the orbit may be an effective treatment in such cases.

## Background

Metastatic tumors in the orbit, especially from gastric cancer, are rare in the USA, Europe, and Japan [1, 2]. In Japan, metastatic tumors account for only 4% of malignant orbital tumors [2]. The most common primary disease sites of orbital metastases are breasts and lungs, and metastasis from gastric cancer is rare [2]. The most common symptoms and signs of orbital metastasis are diplopia and motility disturbance [3]. The time from the diagnosis of gastric cancer to the appearance of ocular signs averaged 25.4 months, and the time from the appearance of ocular signs to death averaged 3.3 months [2]. The way of diagnosis of metastatic tumor in the orbit was imaging studies, that is, computed tomography (CT) and magnetic resonance imaging (MRI) and a fine-needle aspiration biopsy [4].

Here we report a case of metastases from gastric cancer to the medial rectus muscle of the left eye, as demonstrated by the clinical symptoms and CT imaging.

## Case presentation

A 54-year-old Japanese woman visited our ophthalmology department after experiencing proptosis, lid swelling, diplopia, and retro-orbital pain in her left eye lasting for 1 day. She had a medical history of poorly differentiated adenocarcinoma of the stomach, which had metastasized to her ovary and mesentery, diagnosed 2 years earlier. She had undergone four regimen courses of chemotherapy, yet these had failed and she thus received palliative treatment. There were metastases to subcutaneous tissue of her neck and thoracic bone marrow 3 months before her initial visit to our ophthalmic department. She had been admitted to our hospital 5 days previously without symptoms in either eye. She had undergone stenting in her esophagus against eating difficulties but she lived a self-reliant life at home.

**Fig. 1:**
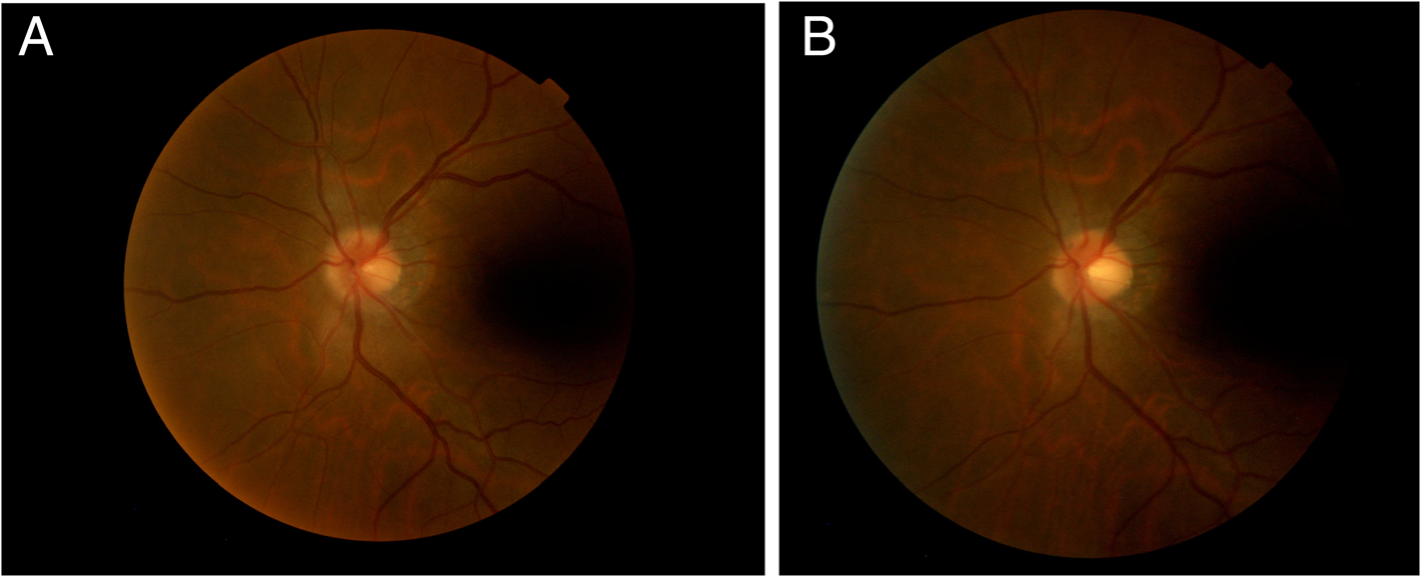
Optic disc edema before and after radiation therapy. **a** Funduscopic examination showed partial optic disc edema in the left eye at the first visit (16 Mar 2018). **b** Optic disc edema had disappeared 6 weeks after radiation therapy (11 May 2018)

At her first visit, an external examination showed lid swelling, red coloration, and proptosis of her left eye. A motility examination revealed an adduction deficit of − 4.0 and an abduction deficit of − 1.0. Ophthalmological examinations revealed a best-corrected visual acuity of 20/20 and an intraocular pressure of 15 mmHg in both eyes. No abnormal findings were found in the anterior segment. Her pupils were equally reactive without any relative afferent pupillary defect. A funduscopic examination showed partial optic disc edema in her left eye (Fig. 1a). No choroidal masses or striae were noted.

**Fig. 2:**
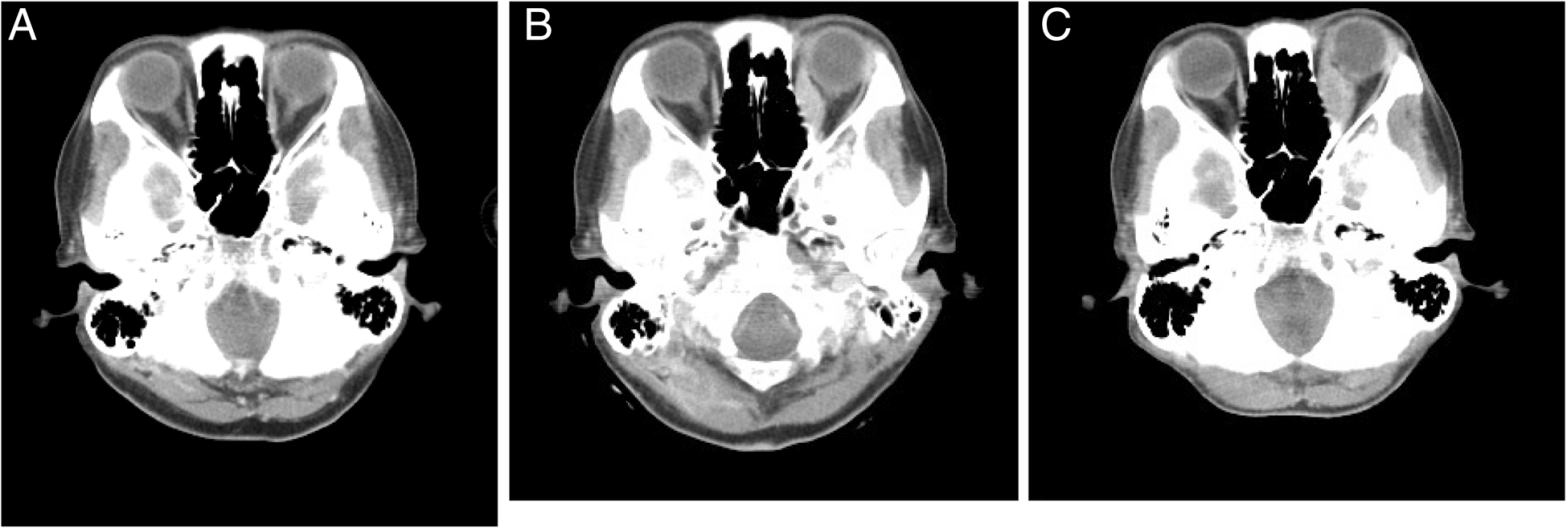
Serial computed tomography images of the left medial rectus muscle. **a** Computed tomography scan showing a normal left medial rectus muscle at 6 months before the first ophthalmological visit (Sep 2017). **b** Computed tomography scan showing enlargement of the left medial rectus muscle at 3 months before the first ophthalmological visit (Dec 2017). **c** Computed tomography scan of the left medical rectus muscle taken 10 days before the first ophthalmological visit (5 Mar 2018)

A CT scan performed 10 days before her initial visit to our ophthalmology department revealed enlargement of the left medial rectus muscle. Retrospectively, similar findings were seen on a CT scan performed 3 months previously, and had worsened in the interim. Yet, a CT scan that had been performed 6 months previously showed no remarkable findings (Fig. 2). There was no enlargement of other extraocular muscles and no swelling or mass lesion in other orbital tissues during the 6 months. So, gastric cancer metastasis to the medial rectus muscle of her left eye was suspected. Radiation therapy for metastasis to the subcutaneous tissue of her neck and thoracic bone marrow was effective; she received a total of 20 Gy/5 courses of radiation therapy to the orbit. A few days after completion of radiation therapy, lid swelling, red coloring, and pain disappeared. Two weeks post-radiation therapy, a motility examination revealed an adduction deficit of − 4.0 and Hertel’s exophthalmometry measurements with a 108-mm base were 14 mm (right eye) and 19 mm (left eye). At 1.5 months post-radiation therapy, a motility examination revealed an adduction deficit of − 2.0 and Hertel’s exophthalmometry measurements (108-mm base) were 14 mm (right eye) and 13 mm (left eye). A posterior ocular segment examination showed a normal left optic disc (Fig. 1b). She died 3 months after her initial presentation to our ophthalmology department.

## Discussion and conclusion

The differential diagnosis of enlarged extraocular muscle includes thyroid orbitopathy, IgG4-related disease, idiopathic orbital inflammatory syndrome, and orbital tumor. In Japan, metastatic tumors account for only 4% of malignant orbital tumors [2]. The most common primary disease sites of orbital metastases are breasts and lungs, and metastasis from gastric cancer is rare [2].

Our patient presented with proptosis, diplopia, lid swelling, and pain in her left eye. Since the orbital area was included in the previously obtained CT scans, it was possible to confirm enlargement of the left internal rectus muscle promptly. In patients with a history of malignancy, metastases to the extraocular muscles must be considered in the differential diagnosis. CT scans showed no inflammation sign of orbital fat or lacrimal gland. In thyroid orbitopathy, isolated enlargement of extraocular muscle is rare. She had no symptoms of retraction of the upper eyelids with lateral flare or eyelid lag on down gaze. When we compared the images from the first ophthalmological visit with those that were taken 3 months previously, the left medial rectus muscle had enlarged markedly, and gastric cancer metastasis was highly likely.

Orbital metastasis is characterized by diplopia, proptosis, pain, and dysmotility [3–5]. Our patient was asymptomatic for 3 months, despite left medial rectus muscle enlargement. Given that proptosis and diplopia would require time to develop, extraocular muscle metastasis may thus be difficult to detect.

Ocular metastases are mainly treated by radiation and chemotherapy [5]. To improve orbital symptoms, palliative radiation therapy was performed for the left medial rectus muscle and rapidly reduced clinical signs. Diplopia improved after radiation therapy, and proptosis was reduced.

The most common complications of radiotherapy to the eyes are cataract, radiation retinopathy, and optic neuropathy [6]. Complication was possible in this case, but life expectancy was thought to be a few months at her first visit to our department, and considering the delay to the appearance of complication, radiation therapy was considered to have merit.

Unfortunately, she died 3 months after her first visit to our department; however, she did not complain of any ocular pain or diplopia after orbit radiation therapy, and radiation therapy contributed to maintenance of her quality of life (QOL).

Extraocular muscle metastasis of gastric cancer is very rare. Symptoms such as diplopia and ocular pain are strong obstacles to QOL. It is difficult to diagnose extraocular muscle metastasis especially before symptoms develop, but it is necessary to follow up on patients with gastric cancer with the possibility of metastases to the orbit in mind.

## Abbreviations

CT: Computed tomography
MRI: Magnetic resonance imaging
QOL: Quality of life
